# Revolutionizing Lithium Metal Anodes With 3D‐Printed Topology‐Optimized Hosts for Enhanced Stability

**DOI:** 10.1002/advs.202521086

**Published:** 2026-03-25

**Authors:** Xin Hu, Yimin Chen, Yun‐Fei Fu, Daoguang Bi, Baozhi Yu, Ying (Ian) Chen

**Affiliations:** ^1^ Institute for Frontier Materials Deakin University Waurn Ponds Victoria Australia; ^2^ College of Mechanical and Electronic Engineering Shandong University of Science and Technology Qingdao Shandong China

**Keywords:** 3D‐printing, additive manufacturing, lithium metal anode, topology optimization

## Abstract

Lithium metal anodes (LMAs) are a key material for next‐generation high‐energy‐density batteries due to their high theoretical capacity and low electrochemical potential. However, their practical use is hindered by issues such as uncontrolled volume expansion and lithium dendrite growth, especially under deep charge/discharge cycling. In this study, a novel LMA host is designed using topology optimization, which directly links structural design with porosity and mechanical stability. Through iterative calculations, reinforced structures are developed that provide support at critical points such as the center, edges, and corners, effectively distributing stress and limiting volume changes during expansion. The topology‐optimized host is fabricated using digital light processing (DLP) 3D printing, whose high resolution and accuracy make it ideal for reproducing complex microstructures. Symmetric cell tests show that the TP host/LMA system maintains stable cycling for over 1000 h under ultra‐high current density (20 mA cm^−^^2^) and high capacity (20 mAh cm^−^^2^). Moreover, in full cells with a LiFePO_4_ cathode, the topology‐structured lithium composite anode retains 95.2% of its capacity after 150 cycles at 5 C. This work demonstrates the potential of combining topology optimization and 3D printing to create ultra‐stable, high‐performance LMAs, and opens a new avenue for the design of advanced batteries.

## Graphical abstract

This study presents a topology‐optimized lithium metal anode host fabricated via digital light processing (DLP) 3D printing. The reinforced microstructure provides mechanical stability, suppresses dendrite growth, and accommodates volume changes. Symmetric and full‐cell tests confirm ultra‐stable cycling under high current density and capacity, demonstrating a promising route for durable, high‐performance lithium metal batteries.

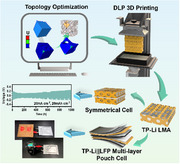

## 1 Introduction

Next‐generation high‐energy‐density lithium‐based batteries, such as lithium‐sulfur or lithium‐air batteries, require significant amounts of lithium metal to achieve high charge/discharge depths in lithium metal anodes (LMAs) at high currents. This imposes new demands on LMAs, including high energy density, long lifespan, and enhanced safety [1, 2, 3, 4]. It is worth noting that most of the current advances are limited to low Li metal utilization, and how to achieve large areal capacity and high current is rarely considered simultaneously [1, 2]. Once the charge/discharge depth of the LMAs is increased, as well as the number of cycles, the uncontrollable volume expansion and dendrite growth of the LMAs are accelerated and amplified under high‐capacity conditions, thus accelerating the failure of the LMAs [1, 2, 5, 6, 7, 8]. Therefore, it remains a great challenge to achieve long‐term cycling stability under high charge/discharge depth. The use of 3D hosts is an effective method to suppress dendrite growth and accommodate the volume changes of LMAs [9, 10]. The high surface area and interconnected porous structure of 3D hosts can reduce local current density and homogenize lithium‐ion flux, ensuring smooth lithium plating/stripping, thereby inhibiting dendrite growth and maintaining lithium's dimensional stability [11, 12]. Moreover, the stable host structure can effectively absorb or mitigate the volume changes of lithium metal during charge and discharge processes, maintaining the integrity of the electrode and preventing the collapse of the internal battery structure and electrolyte leakage, thus extending battery life [13, 14, 15, 16].

Structural design is a key factor influencing the stability of LMA hosts. A well‐designed structure can distribute stress evenly across the host, avoiding localized stress concentrations that could lead to fractures or structural failure [13, 15, 17]. By optimizing the geometry and material distribution of the structure, more uniform stress transfer can be achieved. The host with a structure designed for exceptional buffering capacity can effectively limit the volume changes during lithium deposition, preventing cracking and delamination of the electrode material. To date, several 3D structures, such as porous foam structures (foam copper, foam nickel) and mesh structures (mesh copper, mesh nickel), have been successfully used as 3D hosts for lithium metal anodes (LMAs), significantly improving their lifespan compared to bare LMAs [18, 19, 20]. Additionally, bio‐inspired host structures, such as 3D hosts designed using eggplant cells through carbonization and modification, and 3D hosts modified from the natural channel structure of wood, have also played a crucial role in suppressing lithium dendrites and limiting the volume changes of LMAs [21, 22, 23]. However, whether it's foam structures, mesh structures, or bio‐inspired hosts, due to the inability to precisely customize and control their overall structure leads to severe randomness in the construction of the structure and the arrangement of the pores, and they often suffer from insufficient mechanical strength. During lithium plating and stripping, volumetric expansion of lithium generates mechanical stress at the lithium/host interface. Non‐uniform pore distribution or mechanically weak frameworks can induce localized stress concentration, leading to interfacial instability and uneven lithium deposition. In contrast, mechanically robust hosts with continuous load‐bearing pathways can homogenize stress distribution, thereby indirectly promoting more uniform lithium deposition and suppress dendrite growth. Therefore, it is urgent to develop LMA hosts with superior mechanical stability that can fully accommodate high charge/discharge depth cycling.

Topology optimization is a design method based on mathematical models that optimizes the distribution of materials to achieve the best performance under given conditions [24, 25]. By optimizing material usage and structural shapes, the topology‐optimized structure can meet multiple performance requirements, such as high mechanical strength and high surface area. Topology optimization allows designers to customize designs based on specific application needs. Every aspect of the shape and structure can be precisely controlled through computer simulations to achieve optimal performance [26, 27]. Additionally, 3D printing technology, as an innovative manufacturing method, can produce complex and customized geometries with high precision and detailed reproduction, making it suitable for applications requiring complex designs [28]. In our previous work, we successfully combined topology optimization with 3D printing technology for the first time designing and manufacturing flexible battery electrode structures, which demonstrated exceptional stretchability and battery performance [29]. However, topology optimization has not yet been applied to the design of LMA hosts.

In this work, we combine topology optimization with 3D printing and use finite element analysis (FEA) as structural validation to present for the first time a topological structure host (TP host) for LMAs. Topology optimization directly correlates the porousness and mechanical stability of the host with its structural design. The reinforced structure, developed through iterative calculations, prevents stress concentration during the volume expansion of the LMAs within the host. This enhances the mechanical resilience of the host, effectively constraining the volume change of the LMAs. Complex TP hosts can be accurately and efficiently manufactured using DLP 3D printing technology. Notably, the resulting TP host and LMAs exhibit stable cycling for over 1000 h in symmetric batteries, even under ultra‐high current density (20 mA cm^−2^) and capacity (20 mAh cm^−2^) conditions. Furthermore, in a full battery with a lithium iron phosphate cathode, the topology‐structured lithium composite electrode still has a capacity retention rate of 95.2% (150 cycles) at a high current of 5 C. The emergence of TP hosts presents a valuable solution for ensuring the stable operation of LMAs under conditions of high capacity and high current.

## 2 Results and Discussion

**FIGURE 1:**
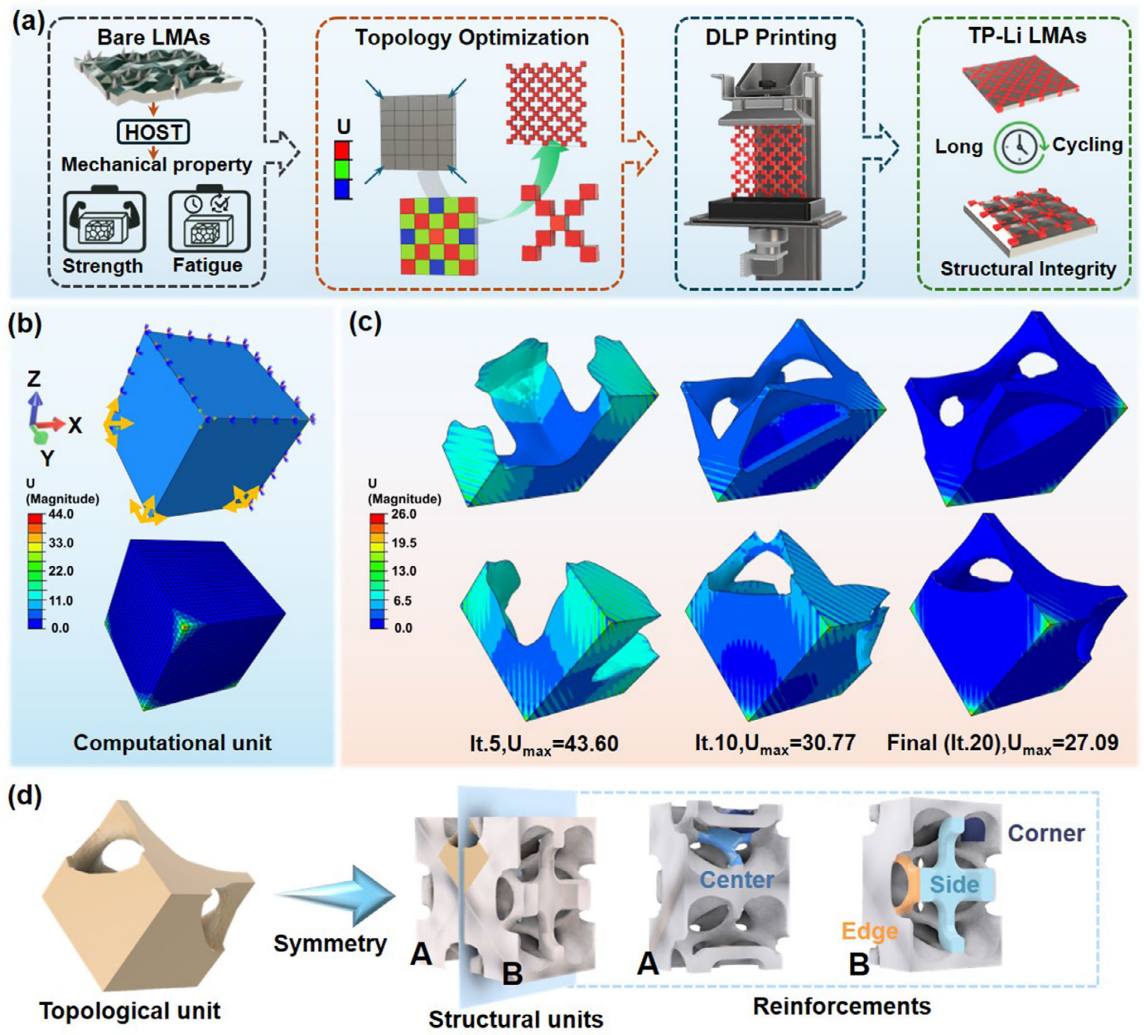
(a) An overview of the proposed workflow for the topology optimization LMA host. (b) Loads on the computational unit and strain energy distribution of the original structure due to load application. (c) Topology optimization results after 5 and 10 iterations and the final optimized structure. (d) Topological units are transformed into structural units by symmetry.

Figure 1a illustrates the design strategy for the LMA hosts using topology optimization. The two main problems present in LMAs are uncontrollable volume expansion and the growth of lithium dendrites. These challenges are translated into mechanical design considerations for topology optimization and are used as a starting point for topology optimization. Next, the mechanical properties are used as optimization objects as the initial conditions for the topology optimization, and the topological units are generated through iterations. The topological units are then arranged in an array to match the battery's use conditions to obtain the final TP host Computer‐Aided Design (CAD) model. Finally, the TP host CAD model is processed using DLP 3D printing technology, and then lithium is deposited inside the host to fabricate composite LMAs. The loading conditions, as shown in Figure 1b, to simulate the forces on the computational unit, involve applying equal pressure loads along the *x*, *y*, and *z* axes at the vertices of a cube while applying symmetric constraints on the faces of the cube. The results of the analysis under these constraints and loading conditions are also depicted in Figure 1b. The design domain for topology optimization is obtained from ABAQUS. According to the results, it can be found that the strain energy sustained by the unit when subjected to stress is mainly concentrated at the vertex and diverges from the fixed point to the surroundings of the computational unit, and the strain energy decreases as it diverges. Based on the results of the analysis of the mechanical properties of Figure 1b, topology optimization calculations are performed on the computational unit. The design domain for structural optimization is based on calculations performed in the Tosca solver module of the commercial finite element analysis software package Abaqus. The built‐in topology optimization capability in Abaqus is based on a density‐based approach, commonly implemented using the Solid Isotropic Material with Penalization (SIMP) method. Figure 1c shows the results for the fifth and 10th iterations and the final result (iteration 20). During the topology optimization process based on preprocessing the computational unit, the material in the center region of the cube gradually decreases. At the same time, the faces along the corners gradually form a reinforcement‐like structure that follows the direction and location of the applied stress loads. As the material is removed and redistributed, the material distribution and structure stabilize, and the material retention reaches 20% after the topology optimization iterations are completed. The change in strain energy with the number of iterations is also shown on Figure 1c. The maximum strain energy starts from 43.60 at iteration 5, and changes to 30.77 at iteration 10. It stabilizes to 27.09 at the last iteration (iteration 20). According to the final topology optimization iteration, a well‐structured topology with excellent stability is obtained (Figure 1d). The iterative analysis of the topology optimization shows that as the number of iterations increases, the strain energy difference between reinforcements calculated by topology optimization, which are iterations decrease, the material distribution stabilizes, and the structure with the lowest strain energy under applied stress loads is formed. The topological unit follows the symmetrical constraints of the mechanical analysis after the symmetry transformation to obtain the structural unit (Figure S1). This structural unit, being composed of topological units, inherits the structure computed by topology optimization, and at the same time, inherits its structural properties. To better study the internal structure of the structural unit, the cross‐section is shown in Figure 1d. It can be observed that there are four types of structures constructed at the center, edges, sides, and corners of the unit. The presence of these four types of reinforcement on the corresponding parts of the unit can disperse the stresses and alleviate the stress concentration when the unit is subjected to external loads, to improve the stability of the structure.

**FIGURE 2:**
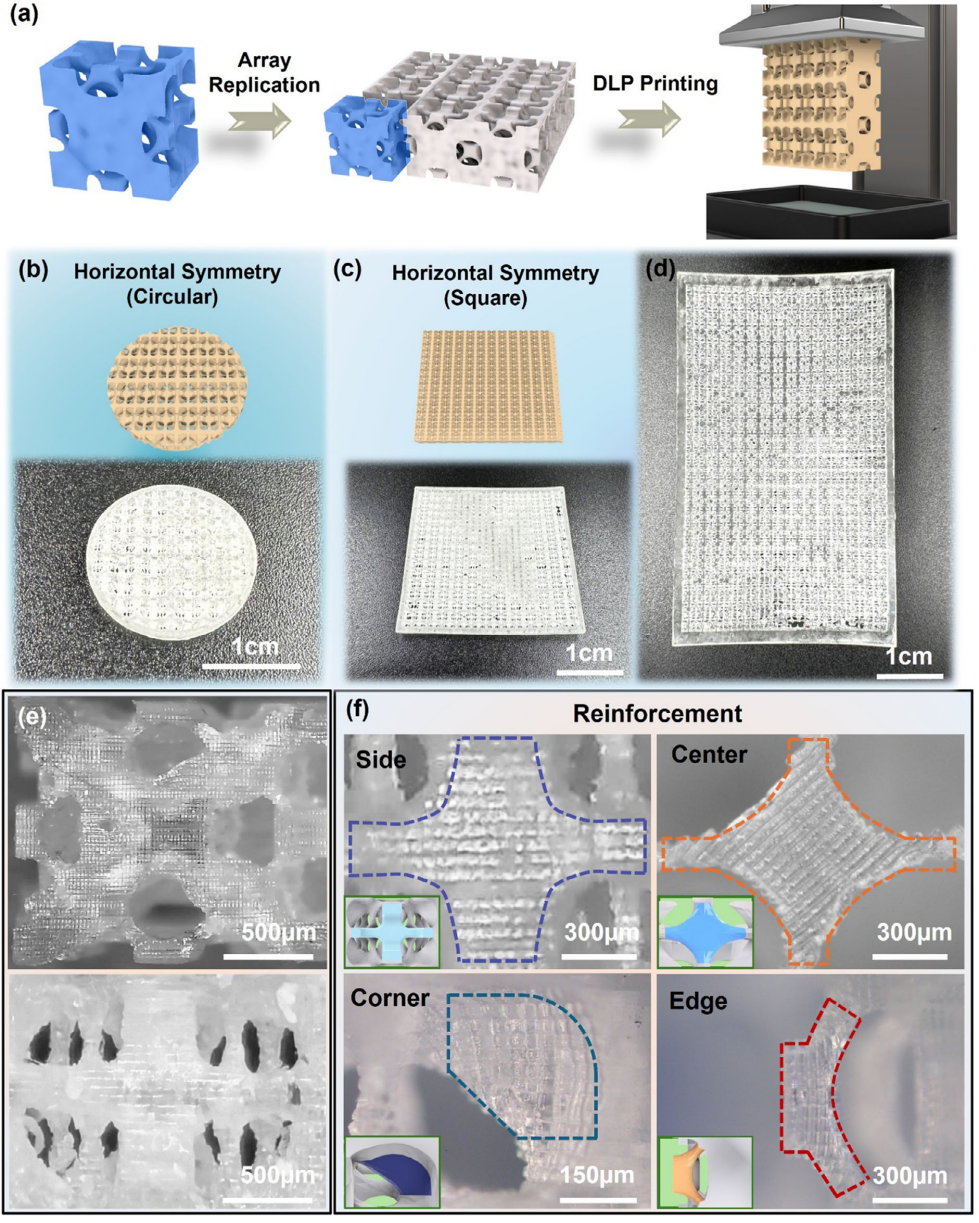
Structural units were constructed into TP hosts after arraying and fabricated by DLP printing (a). The initial TP host is circular for coin cell (b), small‐scale (c) and large‐scale (d) squares for pouch cells. (e) The microscope photographs of the host include a front view and a top view. Microscope photographs of the reinforcements of the TP host, including the side, center, corner, and edge (f).

As shown in Figure 2a, to use the structural unit obtained from the TP host of the LMA, the obtained structural units must first be arrayed and adjusted to produce the final CAD model. The model should conform to the topological structure's mechanical properties. The resulting CAD model was then fabricated by DLP 3D printing. DLP printing uses a digital light projector to cure photosensitive resin layer by layer, enabling high printing accuracy and excellent reproduction of fine structural details. The parts manufactured by DLP printing are highly expressive in detail and have a flat surface, so this technology was selected for the TP host. Figure 2b,c both show the 3D‐printed TP host for coin cells and pouch cells. The dimensions of Figure 2b are (R) 15 mm and (H) 1.5 mm, and the dimensions of Figure 2c are (L) 40 mm, (W) 42 mm, and (H) 1.5 mm for the square LMA host of the pouch cells. Moreover, large‐scale square hosts (L) 58 mm, (W) 95 mm, and (H) 1.5 mm are also allowed to be printed, which opens up the possibility of using the TP host for larger‐scale lithium‐metal batteries (Figure 2d). It can be concluded that the design of the TP host is highly customizable and structurally adjustable. The array transformation and shape modification of the TP host can be adjusted according to the shape requirements of the battery. The modified model still maintains the functionality of the topology and can be successfully fabricated using 3D printing technology. In order to observe the structural integrity and detail reproduction of the mainframe printed using DLP, we used a microscope to observe the front and top surfaces of the host separately, and the microscope images are shown in Figure 2e, where each structural unit was observed to be approximately 1.5 cm in length. Topological units are interconnected to form a complete topological structure network. The parts of the host are very structurally intact, with no breaks or imperfections. Figure 2e also shows a microscope photograph of the top view of the host. It can be found that the edge length of the unit is 1.5 cm and the thickness is 1 cm. Microscope photographs show that the topological LMA host produced by DLP printing technology perfectly reproduces the model and is structurally intact with no collapses or cracks. The microscope photographs (Figure 2f) of the reinforcements of each part with the structural units in the host and the corresponding model images are used to observe whether the respective reinforcing structures are reproduced by the complete printing. The side reinforcement is clearly shown in the microscope photo. The structure of the reinforcement is reproduced completely by DLP technology. The reinforcement has a transverse length of 1.5 mm and a longitudinal length of 1 mm, which provides reinforcement for the side edges and improves the mechanical stability of the host on the side edges. Microscope photographs and model images of the center reinforcement are also shown in Figure 2f. The center of the structural host is 500–600 µm, and the extension of the center to the periphery is about 150–300 µm. Compared with the model photograph of the central reinforcing structure in this figure, it can be found that the host has completely retained the central reinforcing structure in the model. For the edge reinforcement structure, the microscopic photographs show that the width of the center of the structural host is 500 µm, and the width of the extension from the center to the sides is about 150 µm. The edge reinforcement structure of the model is also retained intact in the host. The corner reinforcement structure is clearly shown in the microscope photograph. The structure utilizes a curved surface with a radius of about 300 µm instead of a right angle to strengthen the corner stability. A comparison of its host photo and the model image leads to the conclusion that the corner reinforcement in the model is similarly reproduced intact in the host. Based on Figure 2f, it is concluded that the TP host has flexible structure customization, and the TP host fabricated using DLP printing technology can perfectly reproduce the topological model and the details of the reinforcement in each part.

Stress tests were conducted to confirm the superior mechanical stability of the TP host, and the results were compared with those of the commonly used Mesh structure host (Mesh host). To ensure a fair mechanical comparison, both the TP host and the Mesh host were designed with identical overall dimensions, identical volume fraction (30% material retention), and comparable relative density. All samples were fabricated using the same photocurable resin, identical printing parameters, and identical post‐processing conditions. Therefore, the observed differences in mechanical response arise solely from geometric configuration rather than differences in material composition or density.

**FIGURE 3:**
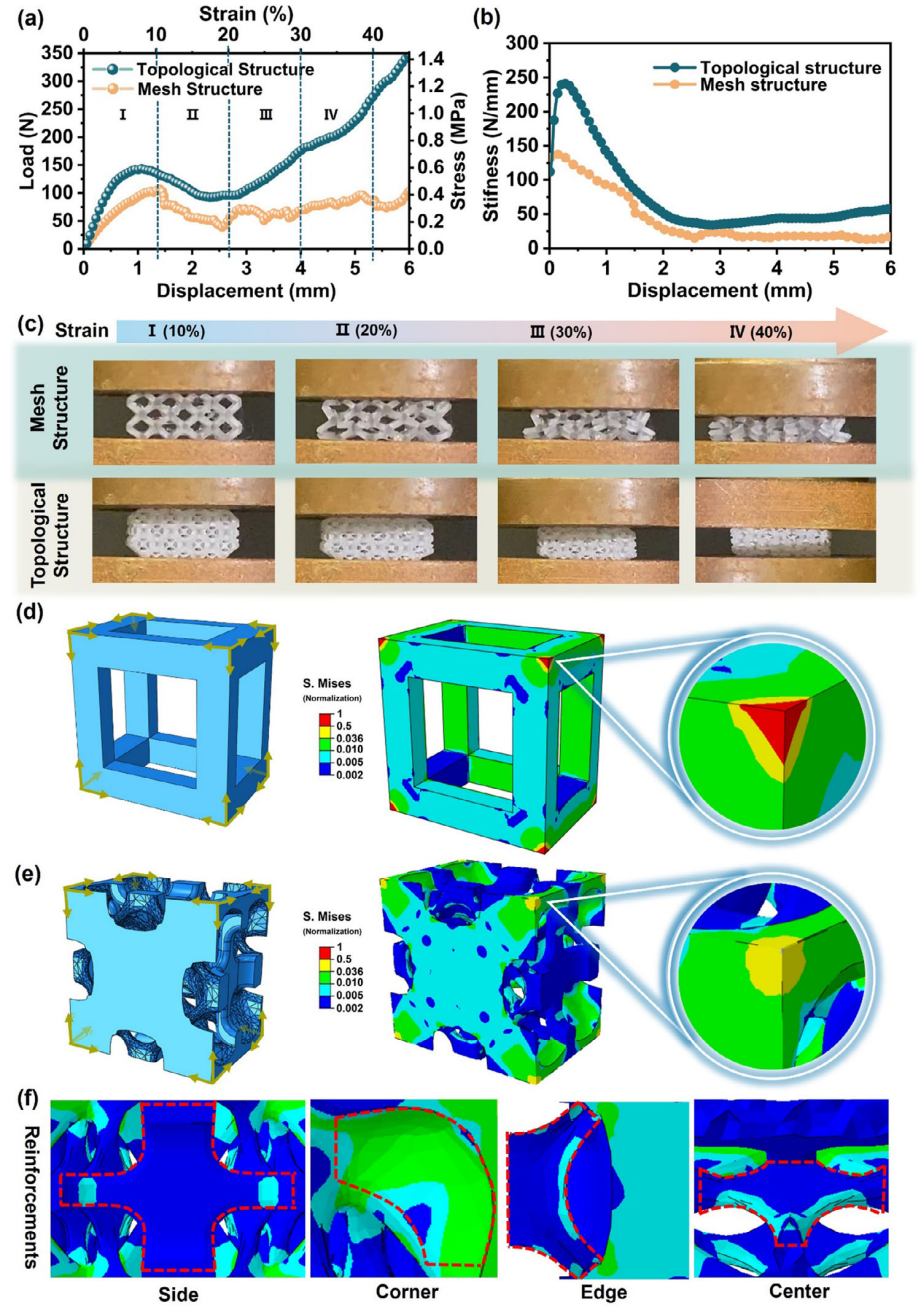
(a) Load‐displacement curve and (b) stiffness–displacement curve of the TP host and Mesh host. (c) The side view of the TP host and Mesh host at marks I, II, III, IV in Figure 3a. FEA of material stress under the same pressure load for Mesh host (d) and TP host (e). Finite element analysis of reinforcements in topological structure under stress load (f). Structural units were passed through the structural host after arraying and were fabricated by DLP printing.

Each 10% strain interval was considered as an observation segment, marked as I, II, III, and IV, respectively (Figure 3a). It is clear that in segment I (strain 0%‐10%), the load‐carrying capacity of both structures starts to decrease, which indicates that both the topological structure and the Mesh structure are starting to fracture. However, at the end of segment I, the TP host withstands a load of 125 N, while the Mesh host withstands only 100 N. This illustrates that within the initial 10% strain, the TP host can bear a greater load than the Mesh host. In segment II (strain 10%–20%), the load‐bearing capacities of both structures continue to decrease, indicating a decline in stability for both structures. However, the load‐bearing capacity of the TP host remains higher than that of the Mesh host. In segment III (strain 20%–30%), both structures experience an increase in load. The load‐bearing capacity of the TP host increases more significantly than that of the Mesh host. At the end of segment III, the TP host withstands a load of 200 N, whereas the Mesh host only bears 70 N. In segment IV (strain 30%–40%), the load‐bearing capacity of the Mesh host stabilizes around 85 N, while the load‐bearing capacity of the TP host continues to rise, reaching 250 N. This experiment demonstrates that a TP host can bear a significantly higher maximum load under the same strain as a Mesh host. Figure 3b shows the changes in stiffness of the TP host and the Mesh host under pressure tests. The curve reveals that the maximum stiffness of the TP host is 250 N mm^−1^ and stabilizes at 65 N mm^−1^, while the maximum stiffness of the Mesh host is 130 N mm^−1^ and stabilizes at 25 N mm^−1^, which is clearly lower than that of the TP host. Figure 3c presents optical images of the structural changes in both host structures during the pressure tests. The Mesh host collapses under pressure and is almost completely destroyed at a strain of 30%. In contrast, the TP host, including the reinforced structures, remains intact even at 40% strain. Therefore, it can be concluded that the TP host has higher mechanical stability and can withstand greater loads than the Mesh host. In order to verify the stress and strain distribution within the structure when the Mesh host and the TP host are subjected to the same load, the FEA was conducted to simulate the stress distribution in the materials under the same pressure load. The mechanical property parameters of the materials used in the FEA simulation were obtained from the ELEGOO resin technical data sheet (Table S1), and the results were normalized for a more intuitive comparison of the stress differences between the two structures. Figure 3d shows the stress distribution in the Mesh host under a pressure of 2000 N. The stress is primarily concentrated at the two vertices where the load is applied. The maximum stress at the vertices is 1 (normalized result), and the area where the structure experiences stress greater than 0.005 (normalized result) accounts for approximately 80% of the overall structure. The results of FEA show that the Mesh host is loaded in each edge in the corresponding direction, so the Mesh host cannot effectively spread the loads applied, which leads to local stress concentration, and the maximum stress applied to the material is large, increasing the risk of structural collapse. Figure 3e illustrates the stress distribution in the TP host under a pressure of 2000 N. The stress is still primarily concentrated at the two vertices where the load is applied. The maximum stress at the vertices is 0.5 (normalized result), which is significantly lower than the 1 (normalized result) in the Mesh host. The area where the structure experiences stress greater than 0.005 (normalized result) accounts for approximately 50%, much lower than the 80% in the Mesh host. It can be observed that, under the same stress load, the maximum stress experienced by the TP host materials is only half that of the Mesh structure, and the overall stress in the structure is lower than that of the Mesh structure hosts. This is because the loads from the outside at various places inside the TP host will be either borne or dispersed by the reinforcement from the outside, thus alleviating the stress concentration phenomenon and thereby enhancing the structural stability of the host. Figure S2 also shows the FEA results of the TP unit and the Mesh unit in front and side views. FEA simulations were also carried out to investigate the stress distribution of the individual reinforcements in the TP host when subjected to loads. Figure 3f illustrates the stress distribution in the center reinforcement of the TP host. The center reinforcement spreads the downward force in four different directions at an angle of 45°, reducing the stress concentration and improving the structure's stability. The edge and corner reinforcements also spread the concentrated forces in different directions, thus strengthening the structure. The stresses in the reinforced structure shown in Figure 3f are all less than 0.036, indicating that the stress dispersion in the reinforced area is excellent and that the reinforcement itself does not introduce new stress concentrations. Figure 3f shows the stress distribution of the TP host reinforcements. The side reinforcement plays the role of reinforcement for the side and less dispersal of self‐imposed loads, the loaded area accounts for about 10% of its volume; the corner reinforcement disperses the load concentrated in the corner through the arc structure, and the loaded area accounts for about 90% of its volume; the edge reinforcement plays a role similar to that of the side reinforcement by redistributing stress along the edge region. The role of the center reinforcement is to spread the load of each part of the TP host at its center and relieve stress concentration. The stresses in the reinforced structure shown in Figure 3f are all less than 0.036, indicating that the stresses in the reinforced area are well dispersed and the reinforcement itself will not bring new stress concentrations.

**FIGURE 4:**
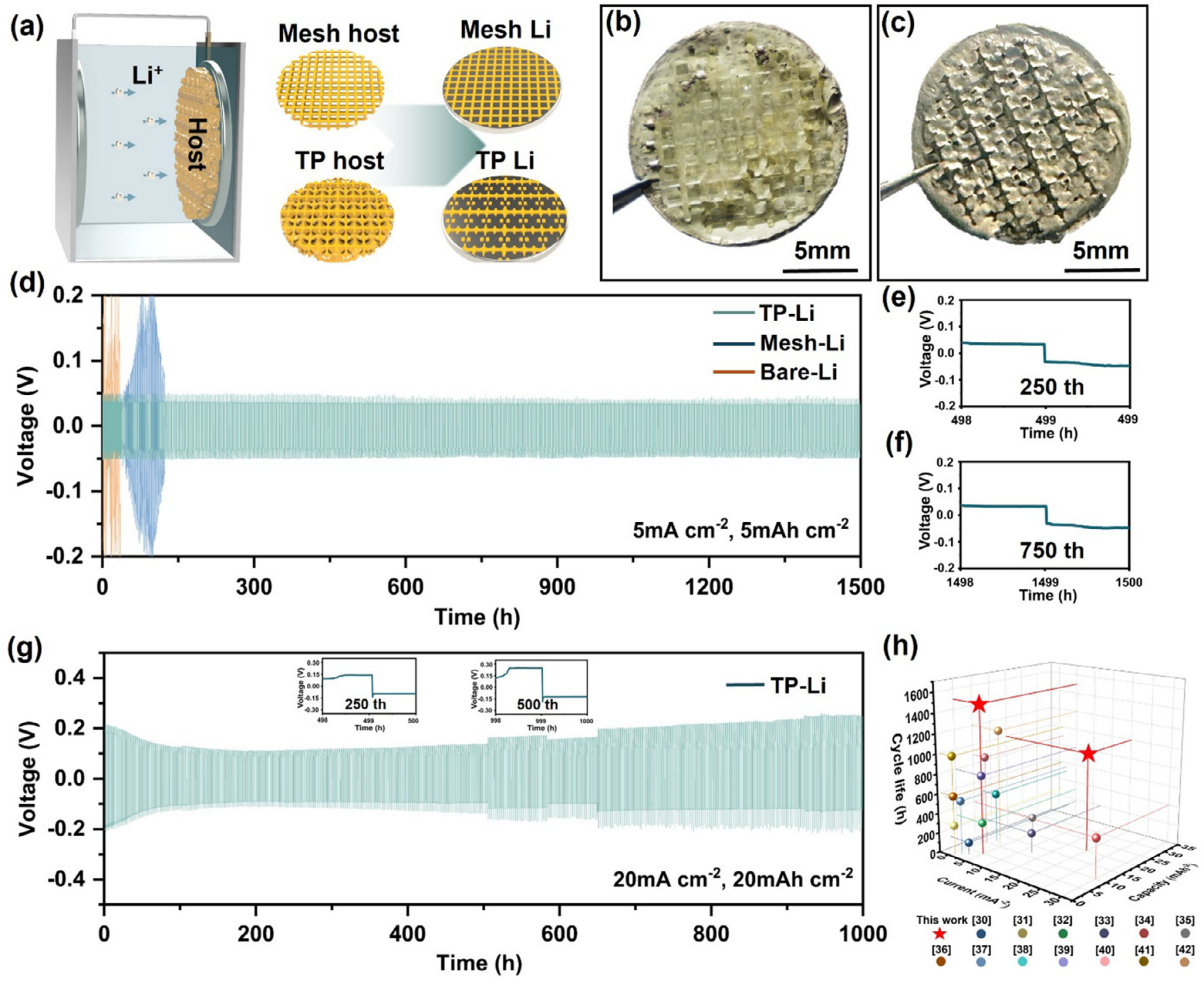
(a) Illustration of the fabrication of Mesh host composite LMAs (Mesh‐Li) and TP host LMAs (TP‐Li) using lithium plating. Optical image of Mesh‐Li (b) and TP‐Li (c). Voltage profiles of bare Li, TP‐Li, and Mesh‐Li based symmetric cells at 5.0 mA cm^−2^ (d). Voltage profile of TP‐Li at 250th cycle (e) and 750th cycle (f). Voltage profiles of bare Li, TP‐Li, and Mesh‐Li based symmetric cells at 20 mA cm^−2^ (g). (h) Comparison of symmetrical battery cycling performance of TP‐Li with previously reported values [30, 31, 32, 33, 34, 35, 36, 37, 38, 39, 40, 41, 42].

The fabrication of Mesh host composite LMA (Mesh‐Li) and TP host composite LMA (TP‐Li) was carried out using lithium plating (Figure 4a). Lithium was pre‐plated on the Mesh host and TP host for 40 h at a current of 10 mA (Figure S3), resulting in the formation of Mesh‐Li and TP‐Li. Figure 4b shows an optical photograph of the Mesh‐Li. Lithium metal was successfully integrated with the Mesh host to form the composite anode. Figure 4c shows an optical photograph of the TP‐Li. Lithium was deposited inside the TP host during electroplating and was well integrated with the host framework. Symmetric cells were assembled and tested under various Li plating/stripping conditions to evaluate the long‐term cycling performance further. For comparison, another symmetric cell was assembled using two identical bare Li. Figure 4d shows the voltage‐time profiles of the symmetric cells composed of TP‐Li, Mesh‐Li, and bare Li at a current density of 5 mA cm^−2^ for a capacity of 5 mAh cm^−2^. Among the three symmetric cells, the TP‐Li showed excellent cycling stability for more than 1500 h and no appreciable voltage fluctuations, the overpotential of TP‐Li was only 0.067 V after 250 cycles (Figure 4e) and 0.0816 V after 750 cycles at a current density of 5 mAh cm^−2^ (Figure 4f). In contrast, the voltage of the battery with the Mesh‐Li was relatively stable at 45 h, but the voltage fluctuated significantly after 45 h. The battery showed a large voltage hysteresis after 100 h (overpotential 0.46 V) and failed after 120 h. The bare‐Li cell showed the largest voltage hysteresis lag (overpotential 0.6 V) and severe fluctuations after 20 h, and the battery failed after 33 h. Electrochemical impedance spectroscopy (EIS) further elucidated the differences between Mesh‐Li and TP‐Li. As shown in Figure S4, impedance spectra of Mesh‐Li (Figure S4a) and TP‐Li (Figure S4b) were recorded after the 10th, 50th, and 70th cycles under high‐stress conditions (5 mA cm^−^^2^, 5 mAh cm^−^^2^). For Mesh‐Li, both charge transfer resistance (Rct) and total impedance increased dramatically with cycling. After 70 cycles, the Nyquist plot showed a steep rise in the real impedance (Z′), a much‐enlarged semicircle, and a near‐vertical spike at low frequency, indicating severe interfacial degradation and hindered ion transport. By contrast, TP‐Li displayed only modest impedance growth, with semicircle diameters remaining relatively stable even after 70 cycles. The low‐frequency region retained a more gradual slope, suggesting efficient Li^+^ diffusion and minimal interfacial deterioration. These findings confirm that TP‐Li's architecture provides improved electrochemical stability by maintaining a robust and conductive electrode–electrolyte interface. To continue to examine the cycling ability of the TP‐Li at higher currents and capacities, the TP‐Li was cycled at 20 mA cm^−2^ and a capacity of 20 mAh cm^−2^ (Figure 4g). The symmetric cell with the TP‐Li is still able to maintain a stable voltage hysteresis for 1000 h. The overpotential of TP‐Li was only 0.2756 V after 250 cycles and 0.421 V after 500 cycles at a current density of 20 mAh cm^−2^. This indicates that TP‐Li is excellent for cycling at higher currents and capacities. This demonstrates that the excellent mechanical properties of the TP‐Li can provide better protection for the LMAs during the volume change caused by Li^+^ deposition and delithiation processes, so that the structure of the LMAs remains intact even at high currents and large capacities, thus enhancing the cycle life of lithium metal batteries. In contrast, because bare lithium is not protected by a host, it fails easily at only 5 mA cm^−2^ and has a capacity of only 5 mAh cm^−2^. Mesh‐Li has a better cycling performance than Bare Li due to the protection of the Mesh host, but their performance is worse than TP‐Li because of their poorer mechanical properties than the TP host. Figure 4h shows a comparison of the capacity and cycling performance of the published composite LMAs [30, 31, 32, 33, 34, 35, 36, 37, 38, 39, 40, 41, 42] To the best of our knowledge, this work demonstrates one of the longest cycling stabilities (1000 h) reported at such a high current density (20 mA cm^−2^) and high areal capacity (20 mAh cm^−2^). It also outperforms many previously reported LMAs stabilized by other methods.

**FIGURE 5:**
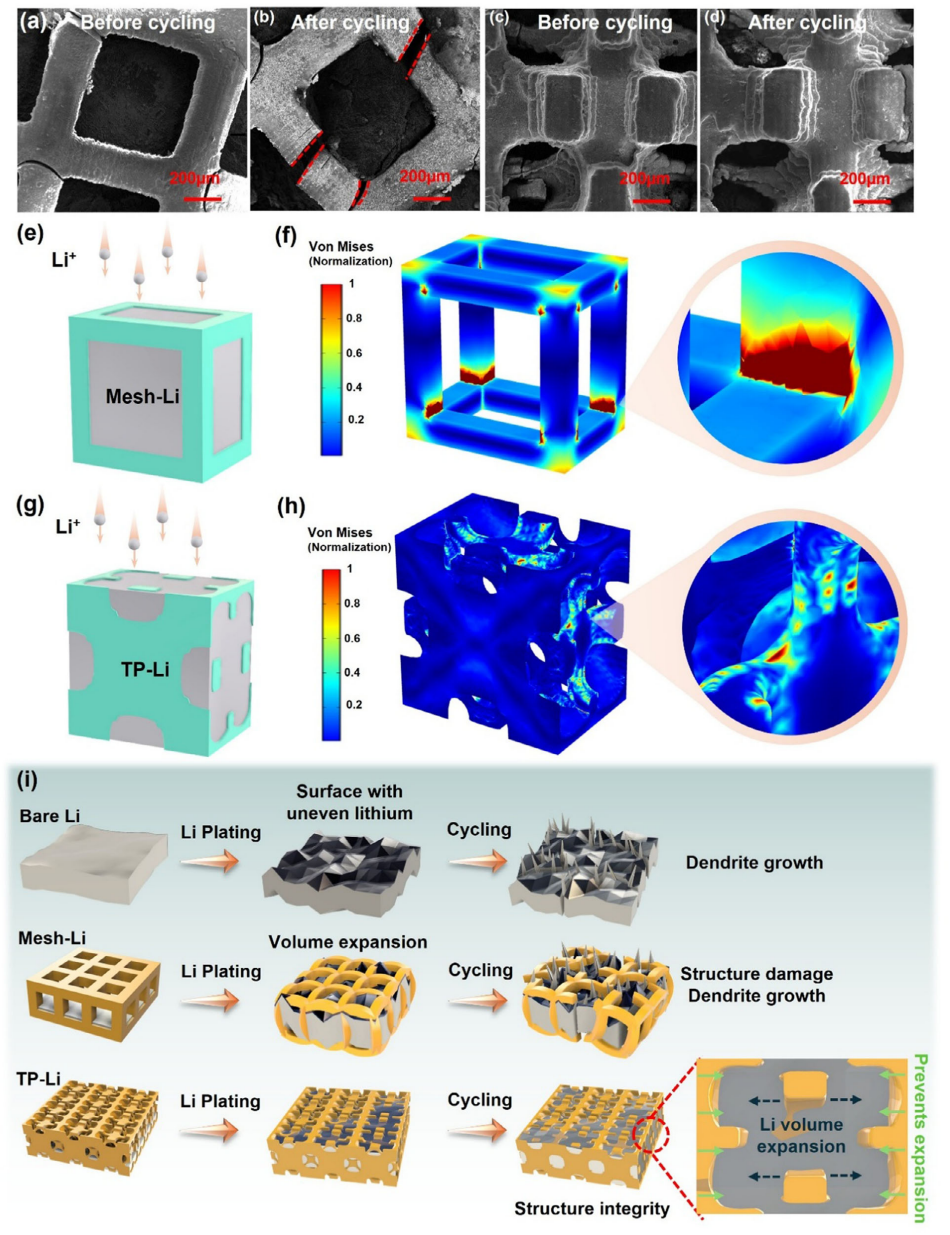
SEM image of Mesh‐Li before (a) and after (b) long cycling. SEM image of TP‐Li before (c) and after (d) long cycling. Schematic (e) and stress results (f) of Multiphysics field simulations of lithium symmetric batteries with Mesh‐Li. Schematic (g) and stress results (h) of Multiphysics field simulations of lithium symmetric batteries with TP‐Li. (i) Schematics of Li plating and behaviour on bare Li, Mesh‐Li, and TP‐Li.

To better study the states of Mesh‐Li and TP‐Li before and after high‐capacity long‐term cycling, we used SEM to observe and compare the two LMAs before and after cycling. Before long‐term cycling, during the initial lithium plating process for fabricating the composite electrode, the volume expansion of lithium caused some minor cracks in the Mesh host of Mesh‐Li, but the overall structure remained intact (Figure 5a). However, after high‐capacity long‐term cycling, the cracks in the Mesh host gradually widened, and the structure collapsed (Figure 5b). In contrast, after the lithium plating stage, the TP host formed TP‐Li with an intact structure and no cracks (Figure 5c); the structure remained intact even after high‐capacity long‐term cycling. The difference between Mesh‐Li and TP‐Li arises because, compared to the Mesh host, the TP host has superior mechanical stability, which can better limit the volume expansion of LMAs during lithium plating and long‐term cycling, thereby enhancing the cycling life of LMAs. To validate the Mesh host and TP host in the lithium‐symmetric battery system, the stress changes in the host during lithium plating were simulated using COMSOL Multiphysics with multi‐physics field simulations and quantified by the mechano‐electrochemical phase‐field model (Table S3). Figure 5e shows the 3D battery model created to simulate the battery operation. In the working state of Mesh‐Li, lithium‐ion deposition within the Mesh host causes substantial expansion and volumetric fluctuation of the lithium phase inside the host. Figure 5f shows the mechanical results of Mesh‐Li. The stress distribution is primarily concentrated at the connection points on the edges of the Mesh host, with the maximum stress being 1 (normalized result). Additionally, the maximum stress is almost entirely distributed at the connection points on the edges of the Mesh host. Figure 5g shows the 3D battery model of the TP‐Li, which is similar to that of the Mesh‐Li 3D battery model. Figure 5h shows the mechanical results of TP‐Li. It can be observed that the stress in the TP host is evenly distributed across the reinforcements of the host, with areas of maximum stress (normalized result of 1) only appearing on isolated reinforcing ribs and occupying a very small proportion, significantly less than that of Mesh‐Li. This is due to the unique reinforcement structure inside the TP host, which can disperse the force of LMAs on the Mesh host uniformly on the sides and inside of the host, thus eliminating the phenomenon of stress concentration. Combining the lithium deposition and cycling processes of the three anodes mentioned above, we can obtain the lithium plating process diagram as shown in Figure 5i. During the lithium deposition process on bare Li, the uneven deposition of lithium results in the accumulation of a large amount of rough lithium on the surface of bare Li, leading to significant volume changes.As cycling continues under high‐current and high‐capacity conditions, these volume changes intensify, leading to the formation of numerous lithium dendrites and dead lithium. This eventually leads to rapid electrolyte depletion until the battery collapses. In contrast, during the lithium deposition process on Mesh‐Li, the Mesh host provides protection, inducing uniform lithium deposition and restricting the volume expansion of Li. The uneven lithium deposition and volume expansion phenomena in Mesh‐Li are significantly improved compared to bare Li. However, during subsequent high‐current and large‐capacity cycling, the volume expansion of LMA intensifies. Due to the limited mechanical stability of the Mesh host, it cannot provide sufficient protection, eventually leading to collapse of the host and the formation of lithium dendrites. In comparison to the above two LMAs, the TP‐Li electrode, due to the outstanding mechanical stability of its TP host, provides excellent protection for the electrode during both the lithium plating stage and high‐current, large‐capacity cycling. The improved electrochemical behavior of the TP‐Li anode can be directly correlated with the topology‐optimized structural features of the host. The continuous load‐bearing pathways generated through topology optimization effectively redistribute the mechanical stress induced by lithium deposition‐related volume expansion, as supported by the finite element analysis results. This stress homogenization mitigates localized mechanical failure and helps maintain interfacial integrity during repeated plating/stripping. In addition, the interconnected internal pores and geometrically constrained framework provide sufficient internal space for lithium accommodation, reducing the tendency for lithium to accumulate at specific surface regions. As a result, lithium deposition is indirectly regulated toward a more spatially uniform behavior, which lowers the effective local current density during cycling. These combined mechanical and geometric effects are reflected electrochemically by reduced polarization, stable voltage profiles, and prolonged cycling stability under high current density and high areal capacity conditions.

**FIGURE 6:**
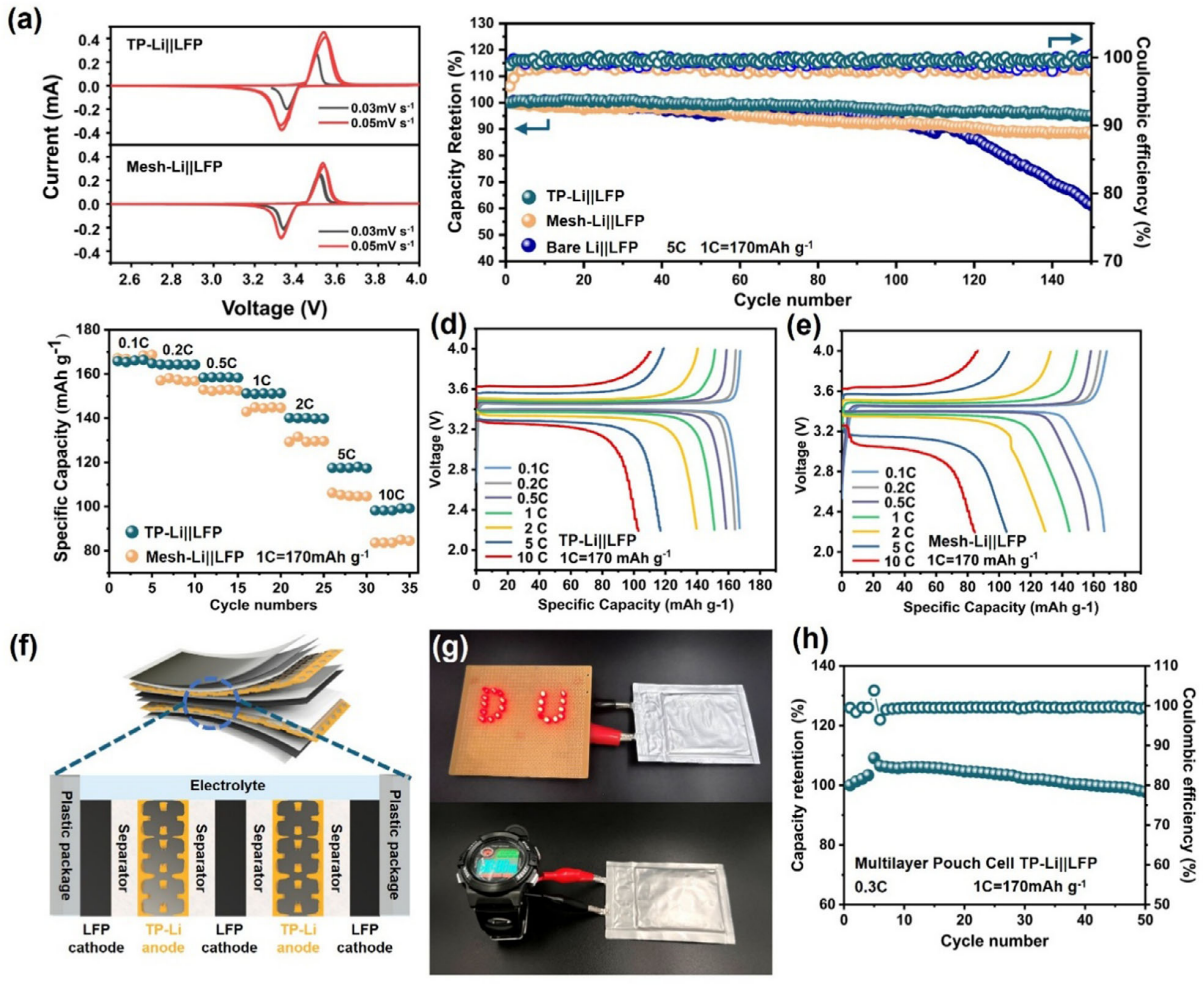
(a) Cyclic voltammetry for TP‐Li||LFP and Mesh||Li‐LFP full cells. (b) Cycling performances and CE of TP‐Li || LFP and Mesh‐Li || LFP full cells at 5C. (c) Rate performances of TP‐Li || LFP and Mesh‐Li || LFP full cells ranging from 0.1 C to 10 C. Charge–discharge profiles of TP‐Li || LFP (d) and Mesh‐Li || LFP (e). (f) Schematic diagram of TP‐Li || LFP multilayer pouch cell structure. (g) Demonstration of a dual‐layer TP‐Li || LFP pouch cell powering LEDs and a digital watch. (h) Cycling performances and CE of TP‐Li || LFP multilayer pouch cell at 0.3 C.

The TP‐Li composite anode was used to assemble a full cell with an LFP (LiFePO_4_) cathode to determine its potential in practical battery applications. In the present configuration, the LFP cathode loading is approximately 3 mg cm^−^^2^ (corresponding to ∼0.45–0.50 mAh cm^−^^2^), and the TP‐Li anode contains excess lithium introduced during the pre‐plating process, resulting in an N/P ratio > 3. This configuration was intentionally adopted to evaluate the structural stability and cycling robustness of the topology‐optimized host under high‐rate conditions, rather than to optimize practical energy density. Future studies will focus on reducing excess lithium and increasing cathode loading toward application‐oriented configurations. The practicality of TP‐Li anodes was tested by constructing complete batteries with LFP cathodes. The Mesh‐Li anode was used as a control for comparison of the same experiments. Figure 6a presents the cyclic voltammetry (CV) curves of half‐cells using TP‐Li and Mesh‐Li as the anode and LFP as the cathode, tested at scan rates of 0.03 and 0.05 mV s^−^^1^. In both cases, distinct redox peaks are observed at approximately 3.45 and 3.30 V, which are characteristic of the Fe^2^^+^ /Fe^3^^+^ redox couple in LFP. This confirms that the electrochemical processes are primarily governed by the intrinsic behavior of the LFP cathode. Notably, the TP‐Li||LFP cell exhibits significantly higher peak current densities and sharper, more symmetric redox peaks at both scan rates, indicating superior electrochemical reversibility and faster interfacial kinetics. Additionally, the smaller peak‐to‐peak voltage separation observed in the TP‐Li system reflects lower polarization and improved reaction efficiency. In contrast, the Mesh‐Li||LFP cell shows broader peaks with larger separation, suggesting increased interfacial resistance and more sluggish charge transfer. Overall, the TP‐Li anode, enabled by a topologically optimized 3D structure, demonstrates enhanced interfacial stability and charge transport capability, clearly outperforming the conventional mesh‐based electrode design and offering a promising approach for high‐performance lithium metal batteries. Figure 6b compares the cycling performance and Coulombic efficiency of full cells assembled with bare‐Li, Mesh‐Li, and TP‐Li anodes paired with LFP cathodes at a high current density of 5 C over 150 cycles. Among the three types of anodes, TP‐Li||LFP delivered the most stable performance, with an initial discharge capacity of 117.3 mAh g^−^^1^ that decreased only slightly to 111.7 mAh g^−^^1^ after 150 cycles, corresponding to an impressive capacity retention of 95.2%. The Coulombic efficiency remained close to 100%, indicating excellent cycling stability and minimal side reactions. In contrast, the Mesh‐Li||LFP cell exhibits an initial capacity of 104.6 mAh g^−^^1^, which drops to 92.7 mAh g^−^^1^ after 150 cycles, yielding a capacity retention of 88.7%, but clearly inferior to that of TP‐Li. Meanwhile, the bare Li||LFP cell suffers from severe capacity fading, with the discharge capacity declining from 114.3 to 69.49 mAh g^−^^1^, resulting in a poor capacity retention of only 60.8%, along with a significantly deteriorated Coulombic efficiency over the course of cycling. These results underscore the superior structural stability and electrochemical reversibility of the TP‐Li anode, which effectively accommodates volume fluctuations and suppresses dendrite formation, making it a highly promising design for high‐rate lithium metal batteries. Figure 6c evaluates the rate performance of TP‐Li||LFP and Mesh‐Li||LFP full cells under various current densities ranging from 0.1 C to 10 C. As shown in Figure 6c, TP‐Li consistently delivers higher specific capacities across all C‐rates compared to Mesh‐Li. At low current densities from 0.1 C to 1 C, both cells maintain relatively high capacities, but the TP‐Li||LFP cell shows slightly better capacity retention. As the current rate increases to 5 C and 10 C, the performance gap becomes more pronounced. The TP‐Li||LFP cell retains a significantly higher capacity (135 mAh g^−^^1^ at 5 C and 120 mAh g^−^^1^ at 10 C), while the Mesh‐Li||LFP exhibits a sharp capacity drop (120 mAh g^−^^1^ at 5 C and 80 mAh g^−^^1^ at 10 C). The corresponding charge‐discharge voltage profiles in Figure 6d,e further confirm these trends. TP‐Li||LFP (Figure 6d) shows flatter and more stable voltage plateaus with lower polarization across all tested C‐rates, even at 10 C, indicating superior reaction kinetics and interfacial stability. In contrast, Mesh‐Li||LFP (Figure 6e) suffers from increased polarization and reduced plateau sharpness at higher rates, reflecting poorer ionic/electronic transport and more severe voltage hysteresis. These results demonstrate that the topologically optimized TP‐Li anode offers enhanced rate capability, lower internal resistance and improved structural integrity under fast charging/discharging conditions, clearly outperforming the conventional mesh‐structured Li anode. Figure 6f illustrates the structural design, practical application, and electrochemical performance of a multilayer pouch cell constructed with a TP‐Li anode. The cell is composed of multiple stacked units of TP‐Li as the anode and LFP as the cathode, separated by electrolyte‐soaked separators and sealed within a plastic package. This layered architecture allows for increased energy density and is compatible with scalable and practical battery configurations. Figure 6g shows real photographs of the assembled multilayer pouch cell and its operational demonstration. The upper image displays the pouch cell successfully powering an LED array shaped into the letters “DU,” while the lower image shows the cell running a digital wristwatch, indicating its reliable output capability and application viability in real electronic devices. Figure 6h presents the cycling performance of the multilayer TP‐Li||LFP pouch cell at a current density of 0.3 C. After 50 cycles, the discharge capacity changed from the initial 161.2 to 160.4 mAh g^−1^, with a capacity retention of 99.53%, highlighting high reversibility and minimal side reactions during charge/discharge processes. Overall, this figure demonstrates that the topologically engineered TP‐Li anode enables the fabrication of high‐performance multilayer pouch cells with robust structural integrity, reliable practical operation, and outstanding long‐term electrochemical stability, emphasizing its strong potential for real‐world energy storage applications.

## 3 Conclusion

In summary, we have designed and fabricated a TP host with excellent mechanical stability for stable lithium plating/stripping by combining topology optimization design and 3D printing. This host uses topology optimization to integrate mechanical stability and porosity into the structural design, and iterative calculations to generate the optimal material distribution, and uses DLP 3D printing technology to achieve high‐precision manufacturing of structures. Compared to conventional planar lithium foils and Mesh hosts, the reinforcement features generated through topology optimization can effectively redistribute stress, greatly reduce the stress concentration phenomenon generated when the host is loaded, enhance the mechanical stability of the host, and improve the cycle life of the LMAs. As a result, TP‐Li was able to achieve stable cycling up to 1000 h with low hysteresis even at a high current density of 20 mA cm^−2^. Moreover, in full cells paired with the LFP cathode, the topologically engineered lithium composite anode exhibits excellent cycling stability, retaining 95.2% of its initial capacity after 150 cycles at a high current rate of 5 C. The strategy of using topological structures and 3D printing techniques to design and fabricate hosts for LMAs to guide the stripping/plating process and limit the volume change can provide insights for further research in terms of framework design for high‐capacity, dendrite‐free LMAs.

## 4 Experimental Section

### 4.1 DLP 3D Printing Host Preparation

Photocurable resin (Elegoo Standard Photopolymer Resin Translucent) was purchased from Amazon.com and used as received. A DLP 3D printer (Flashforge Hunter) has a resolution of 50 µm in all directions (x, y, and z). The initial model cells were derived from the topology design in ABAQUS / TOSCA (SIMULIA). The structural array was then optimized using FUSION 360 (Autodesk) and sliced using Flashforge DLP Printer software. The duration of the UV exposure was 35 s for the first layer and 2 s for the rest. The retraction velocity of the platform was set at 80 mm min^−1^. After DLP printing, the as‐printed structures were carefully removed from the build platform and rinsed with ethanol to eliminate residual uncured resin from the surface and internal pores. The cleaned samples were then dried under ambient conditions, followed by UV post‐curing to ensure complete polymerization of the photocurable resin. UV post‐curing was performed using a commercial UV curing chamber under controlled conditions to enhance the mechanical integrity and dimensional stability of the printed frameworks. This post‐processing procedure was applied consistently to all DLP‐printed samples prior to subsequent characterization and electrochemical testing.

### 4.2 Topology Optimization

Topology optimization was performed using the Abaqus (SIMULIA) FEA software in combination with the Tosca module. The input elastic properties of the material were taken from the ELEGOO resin Technical Data Sheet (Table S2). The algorithm used was the Solid Isotropic Material with Penalization (SIMP) method, with a typical penalty factor of three. In the density assignment, the minimum density was set to 0.001, the maximum density was set to 1, and the maximum change per design iteration was set to 0.25. The convergence condition for the function is a volume retention rate of 30%.

### 4.3 Finite Element Analysis

The deformation behavior of the topology‐optimized host and the square mesh host was modelled using 3D finite element analysis in ABAQUS/CAE 2020. The input mechanical properties of the material were taken from the ELEGOO resin technical data sheet and the models were discretized using four‐node shell elements with a refined mesh. The global Mesh size was approximately 0.05 to ensure Mesh quality.

### 4.4 Coupled Electrochemical–Mechanical Multiphysics Simulation

Finite‐element simulations were performed using COMSOL Multiphysics 6.0 to investigate the mechanical stress evolution in the host structure during lithium plating processes. The model coupled Deformed Geometry, Tertiary Current Distribution, and Solid Mechanics modules to account for the interplay between electrochemical reactions, current transport, and mechanically induced deformation. The computational domain was discretized in COMSOL Multiphysics using a free tetrahedral mesh with a physics‐controlled Normal element size. The maximum and minimum element sizes were set to 2.9 and 0.522 µm, respectively, with a maximum growth rate of 1.5 to ensure smooth mesh transitions. Curvature refinement (factor 0.6) and narrow‐region resolution (0.5) were applied, and mesh smoothing was performed with four optimization iterations to improve numerical stability. A galvanostatic condition was applied at the electrode boundary with an average inward current density of 20 mA cm^−2^, and the initial boundary potential was set to −0.3 V. Interfacial kinetics were described by the Butler–Volmer model with an equilibrium potential of 0 V, exchange current density of 50 mA cm^−2^, and anodic transfer coefficient α_a_ = 0.5. The coupling between electrochemical transport and mechanical deformation was implemented using the Deformed Electrode Surface interface in the simulation. This feature links the evolving electrode geometry with the Tertiary Current Distribution (Nernst–Planck formulation) to account for plating‐induced morphological changes. The moving‐boundary smoothing scheme was enabled to ensure numerical stability during the deformation of the electrode surface. Table S3 further explains the parameters and calculation methods.

### 4.5 Material Characterization

An optical microscope (Olympus DP27) was used to observe and photograph the main and top views of the TP host, as well as details of the reinforcement structure of each section. The microstructure of the 3D‐printed electrodes was characterized using field emission scanning electron microscopy (FE‐SEM, SUPRA 55VP, ZEISS). X‐ray diffraction spectroscopy (XRD, X'pert Powder, Panalytical) was used to analyse the composition of the 3D‐printed electrode material. Uniaxial pressure measurements assessed the prepared electrode host's mechanical stability (UTM, Instron 30 KN, Instron). The strain rate was 0.15 mm min^−1^. Load and displacement data were acquired at 0.5 s intervals. Thermogravimetric analysis (Netzsch TG 209F1 Libra) was performed to analyze the thermal stability of the prepared 3D‐printed electrodes. In TGA measurements, the samples were heated from 25°C to 1000°C in a nitrogen atmosphere at a heating rate of 10°C min^−1^.

### 4.6 Electrochemical Characterization

The coin cells and pouch cells used for electrochemical testing were assembled in an argon‐filled glovebox, with oxygen and moisture levels below 1 ppm (Vigor Tech). For the preparation of TP‐Li and Mesh‐Li, 2032‐type coin cells were used, with polypropylene film (Celgard 2500) as the separator, and 1 m LiTFSI with 2 wt% LiNO_3_ additive dissolved in (DOL)/(DME) (1:1, v/v) ether‐based electrolyte as the electrolyte. For the preparation of TP ‐Li and Mesh‐Li, 2032‐type coin cells were assembled using lithium foilas the counter/reference electrode and the printed host as the working electrode. After plating lithium for 40 h at a current of 10 mA (as shown in Figure 3), the cells were disassembled to obtain TP‐Li or Mesh‐Li. For symmetric cell testing, 2032‐type coin cells were used, with polypropylene film (Celgard 2500) as the separator, and 1 M LiTFSI with 2 wt.% LiNO_3_ additive dissolved in (DOL)/(DME) (1:1, v/v) ether‐based electrolyte as the electrolyte. Both the cathode and anode consisted of TP‐Li or Mesh‐Li. For full cell testing, 2025‐type coin cells were used, with an LFP as the cathode, with an areal loading of 3 mg cm^−^^2^. Polypropylene film (Celgard 3501) was used as the separator, and the liquid electrolyte was 1 m LiPF_6_ dissolved in a mixture of ethylene carbonate (EC) and diethyl carbonate (DEC) (in a 1:1 volume ratio). TP‐Li or Mesh‐Li as the anode. Multi‐layer pouch cells use the same type of separator and electrolyte as coin cells. Battery cycling and C‐rate tests were performed using a cell test system (CT2001A, LANHE).

## Funding

Australian Research Council under the ARC Research Hub for Safe and Reliable Energy (IH200100035).

## Conflicts of Interest

The authors declare no conflicts of interest.

## Supporting information




**Supporting File**: advs74949‐sup‐0001‐SuppMat.docx.

## Data Availability

The data supporting this article has been included as part of the SI. All the original data can be obtained from the corresponding authors upon reasonable request.
