# Supplementary material for: Revolutionizing Lithium Metal Anodes With 3D‐Printed Topology‐Optimized Hosts for Enhanced Stability
Source: Adv Sci (Weinh). 2026 Mar 25;13(32):e21086. doi: 10.1002/advs.202521086 (PMC13252640; doi:10.1002/advs.202521086)
Supplement: Supplementary file 1 — Supporting File: advs74949‐sup‐0001‐SuppMat.docx. [file ADVS-13-e21086-s001.docx]

Supporting Information

Revolutionizing Lithium Metal Anodes with 3D-Printed Topology-Optimized Hosts for Enhanced Stability.

Xin Hu, Yimin Chen, Yun-Fei Fu,Daoguang Bi, Baozhi Yu*, Ying (Ian) Chen*,

X. Hu, Y. Chen, D. G. Bi. Dr. B. Z. Yu, Prof. Y. Chen

Institute for Frontier Materials, Deakin University, 75 Pigdons Road, Waurn Ponds, Victoria 3216, Australia
E-mail: baozhi.yu@deakin.edu.au; ian.chen@deakin.edu.au

Prof. Y. F. Fu,
College of Mechanical and Electronic Engineering, Shandong University of Science and Technology, 579 Qianwan'gang Rd, Huangdao, Qingdao, Shandong, China, 266590.


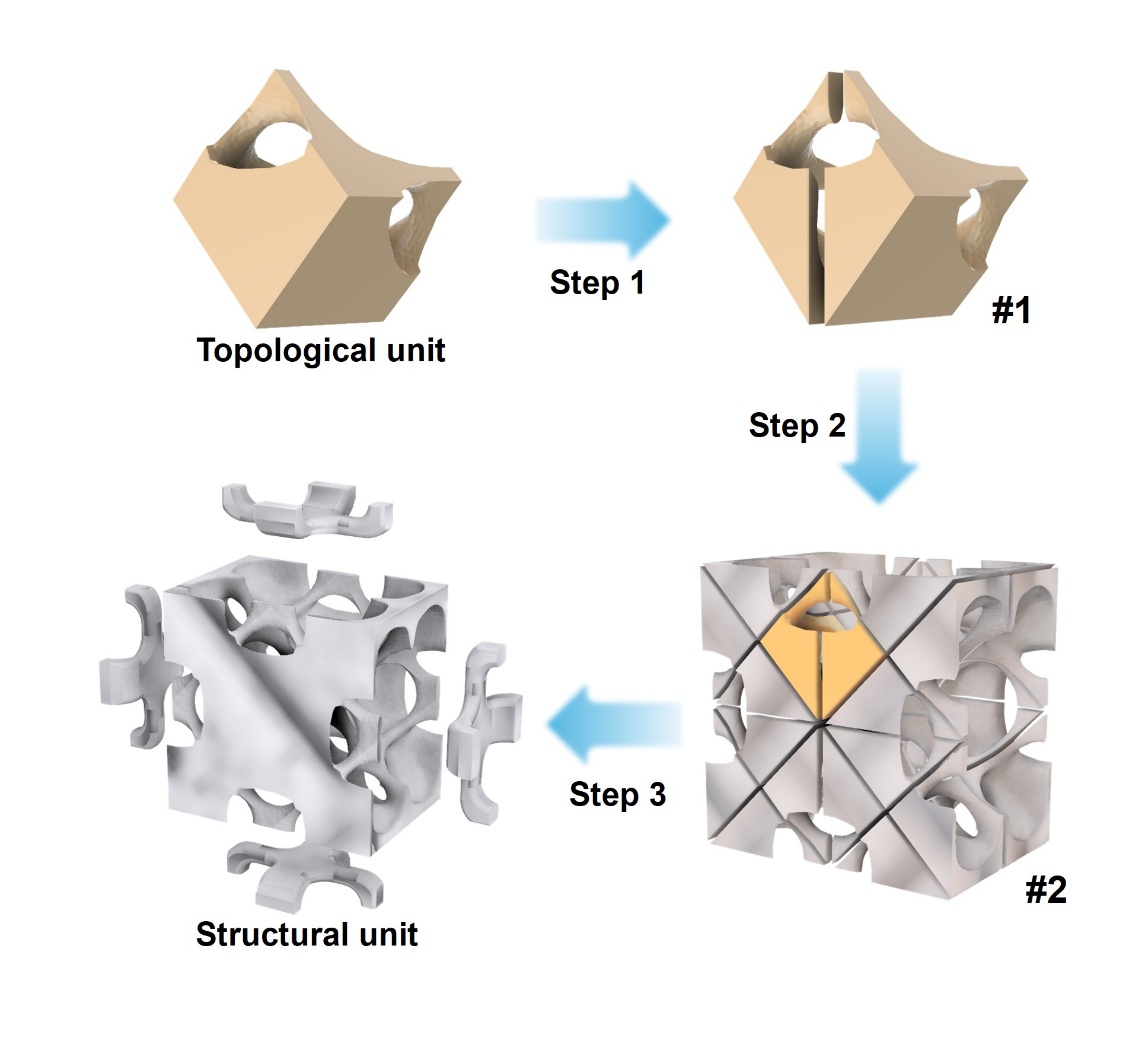


**Figure S1.** The transformation process from the topology unit to the structural unit.

Figure S1 illustrates the transformation process from topological units to structural units. This process is divided into three steps. In the first step, the topological unit is symmetrically split from the center, and the resulting structure after the split is designated as transformation unit #1. In the second step, the units are arranged symmetrically, leading to the formation of unit #2. In the third step, a cross-reinforcement structure is built around the four edges of unit #2, ultimately forming the structural unit.


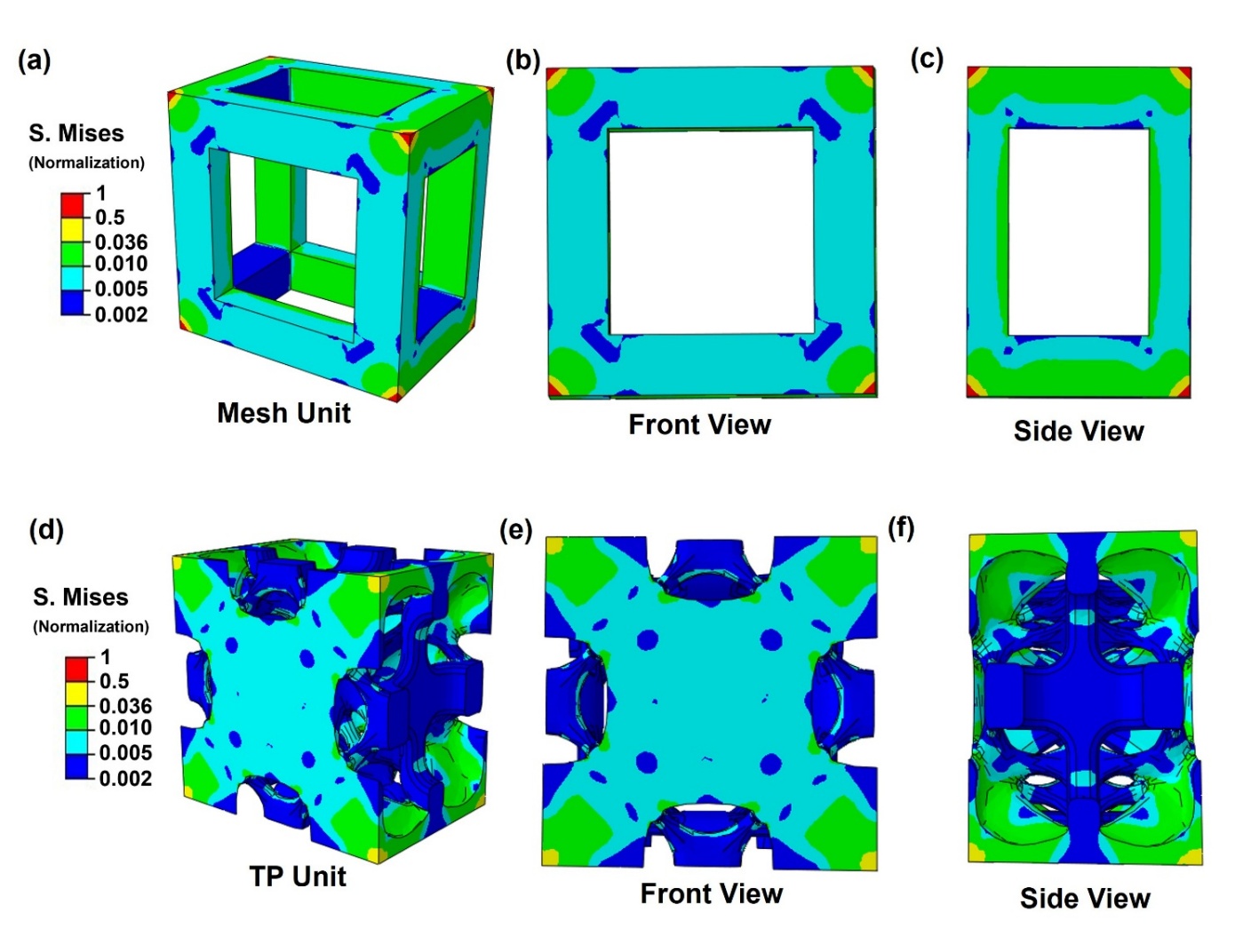


**Figure S2.** FEA results for the front view and side view of the TP unit and the Mesh unit.

Figure S2 shows the FEA results of the front and side views of the TP unit and Mesh unit. By observing the front and top views, it is clear that the maximum stresses in both the TP unit and the Mesh unit are mainly concentrated at the two vertices where the loads are applied. The maximum stress at the vertices of the Mesh unit is 1 (normalized result), and the maximum stress at the vertices of the TP unit is 0.5 (normalized result), which is significantly lower than that of the Mesh unit, which is 1 (normalized result).


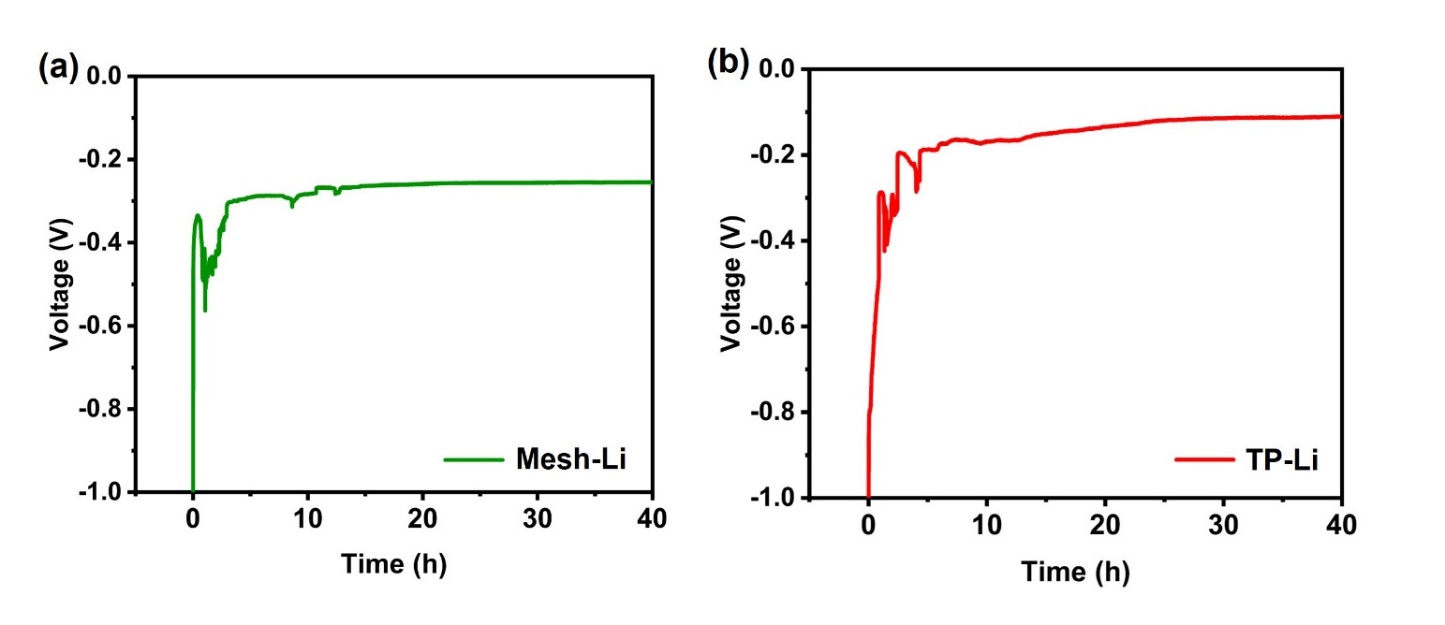


**Figure S3.** Voltage profiles of Mesh-Li (a) and TP-Li (b) during the lithium plating stage.

Figure S3 shows the pre-lithiation voltage curves of TP-Li and Mesh-Li, respectively. The process was carried out under a current of 10 mA for 40 hours, with a total lithium deposition amount of 400 mAh. The voltage of TP-Li stabilized at -0.11V, while the voltage of Mesh-Li stabilized at -0.21 V.

**
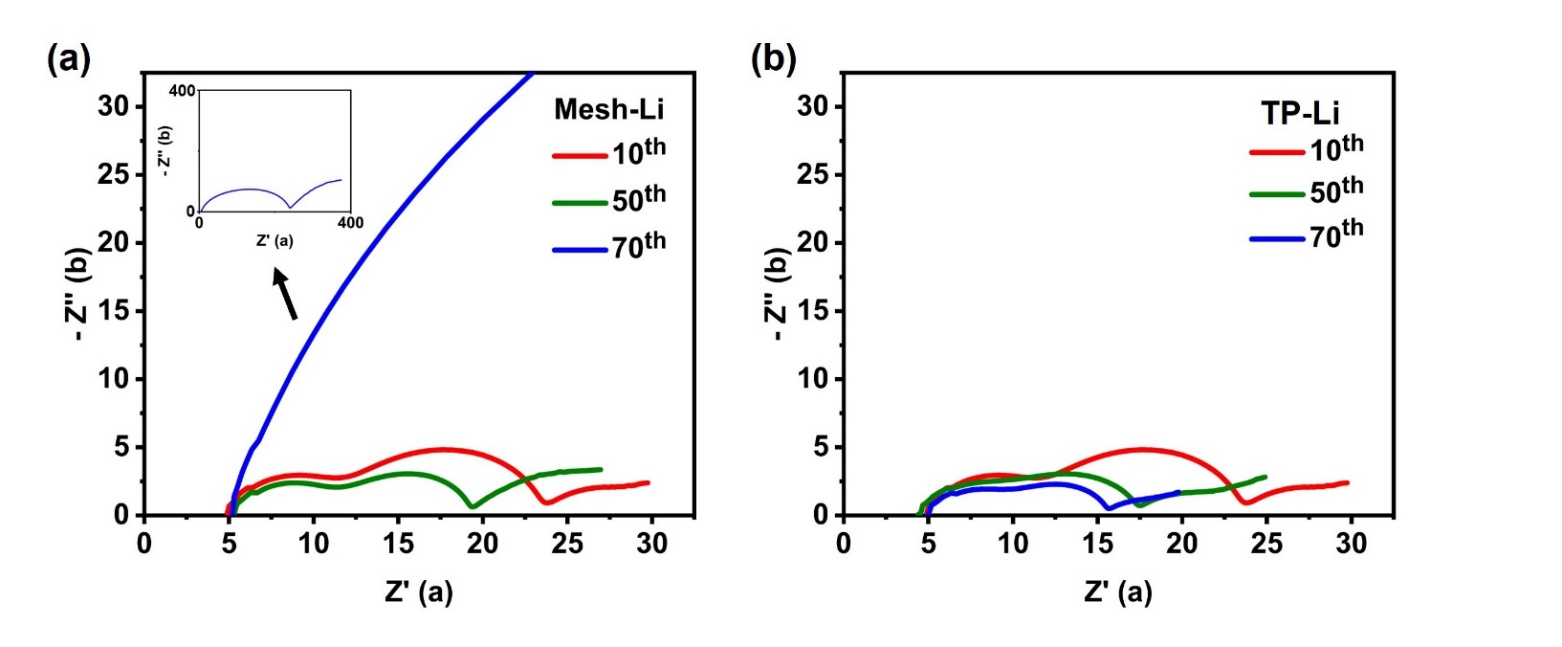
**

**Figure S4.** Nyquist plots of Mesh-Li symmetric cells (a) and TP-Li symmetric cells (b) after 10 cycles,50 cycles, and 70 cycles.

The Nyquist plots corresponding to the EIS results at different cycling numbers under 5 mA cm⁻² and 5 mAh cm⁻² are shown in Figure S4a and Figure s4b. The semicircle in the high-frequency region is related to the interfacial charge transfer resistance (R _ct_). For the cells using Mesh-Li, the R _ct_ value increases from 23.8 Ω at the 10th cycle to 236.8 Ω at the 70th cycle, which is mainly attributed to the continuous growth of lithium dendrites, the volumetric expansion of the lithium metal anode, and the fracture of the Mesh framework. In contrast, for the cells using TP-Li (Figure S4b), the diameter of the semicircle gradually decreases from 23.8 Ω at the 10th cycle to 15.5 Ω at the 70th cycle, indicating a reduction and stabilization of interfacial charge transfer resistance. This can be attributed to the stabilizing effect of the TP framework on the lithium metal anode.


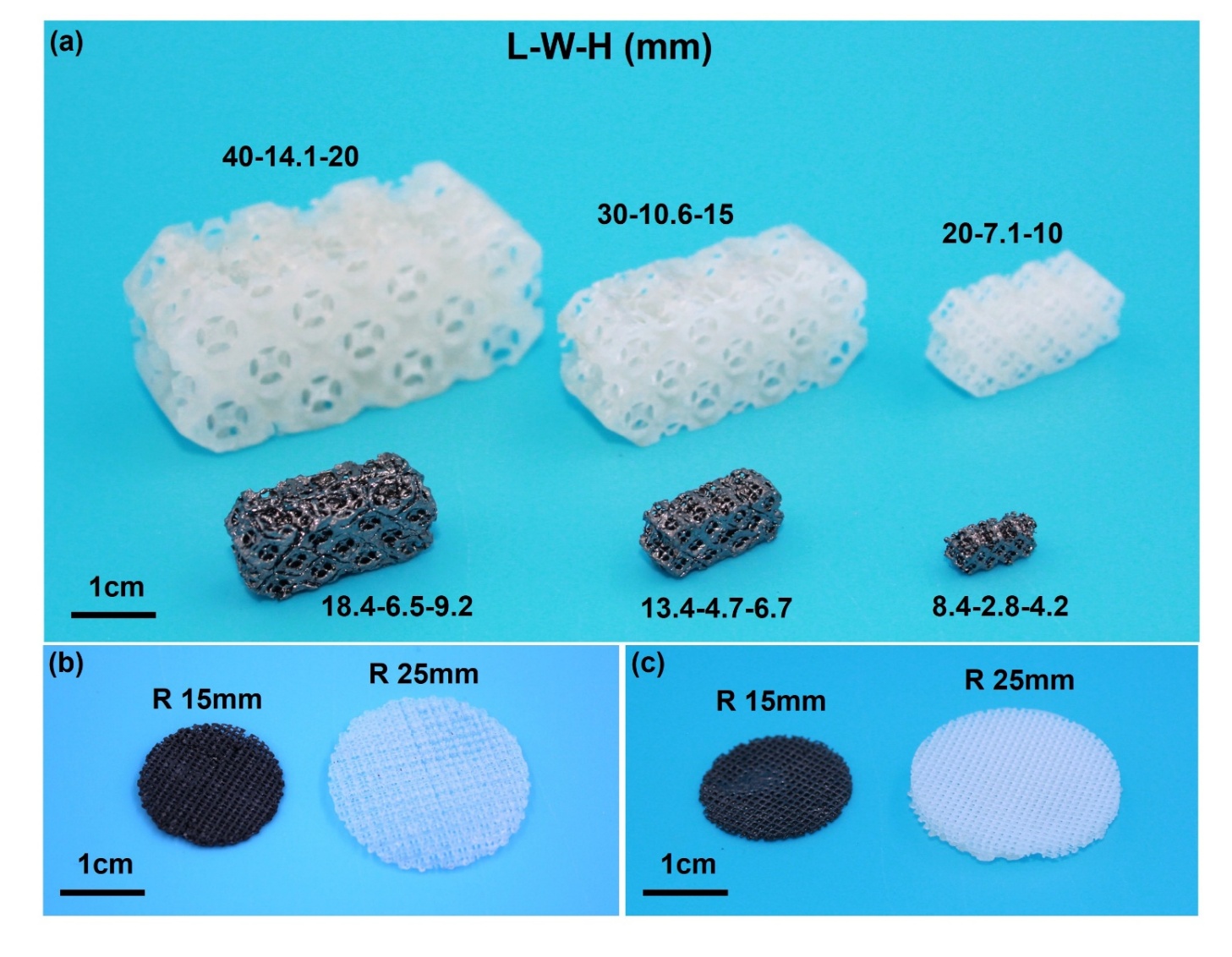


**Figure S5.** 3D-printed photocured resin and its corresponding carbon electrodes. (a) Optical images of printed and carbonized skeletons with topology unit side lengths of 9 mm, 6 mm, and 3 mm. Coin cell topological structure (b) and Mesh structure (c) of printed and carbonized skeletons.

Topological structure and Mesh-structured 3D-printed frames were used as conductive frames by carbonation. The tube furnace was evacuated at 15 Pa throughout the carbonization process. The resin was pyrolyzed under 15 Pa, first at 400 °C for 4 h and then at 1000 °C for an additional 4h. Throughout the process, the temperature was 10 °C min^-1^. The samples are labeled "L-W-H", referring to the length, width, and height of each topological 3D model in this study: 40-14.1-20, 30-10.6-15, and 20-7.1-10, as shown in Figure S5a. The white micro-lattice is a sample of resin isolated from the base, replicating the structural features that appear in the original 3D model. The micro-lattice remained intact during pyrolysis despite a linear shrinkage of approximately 55% in each direction. A comparison between the topologically structured disc model and the lattice-structured disc model before and after carbonization is shown in Figure S5(b) and (c). The linear shrinkage in each direction after carbonization is approximately 45% and the structure remains intact during pyrolysis. The excellent structure retention properties of the thermal structure offer the possibility of direct use as a conductive framework for electrodes.


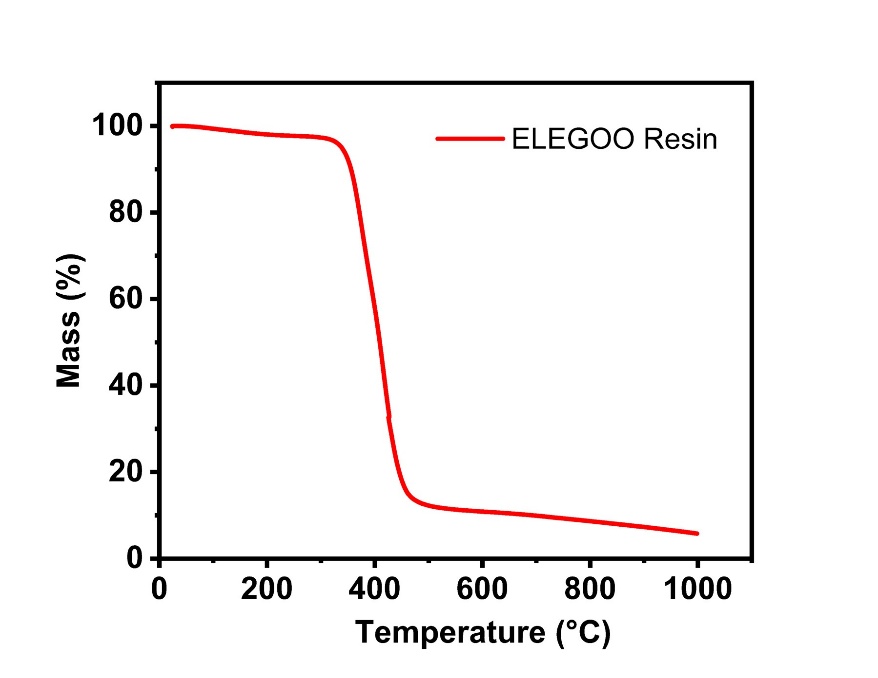


**Figure S6.** Thermogravimetric (TGA) curve of a 3D-printed skeleton.

Figure S6 shows the weight change of the ELEGOO material from 20 ℃ to 1000 ℃. The figure shows that the carbonization process is evident when the material is 350-450 ℃. After carbonization, the weight is about 10 % of the original weight.


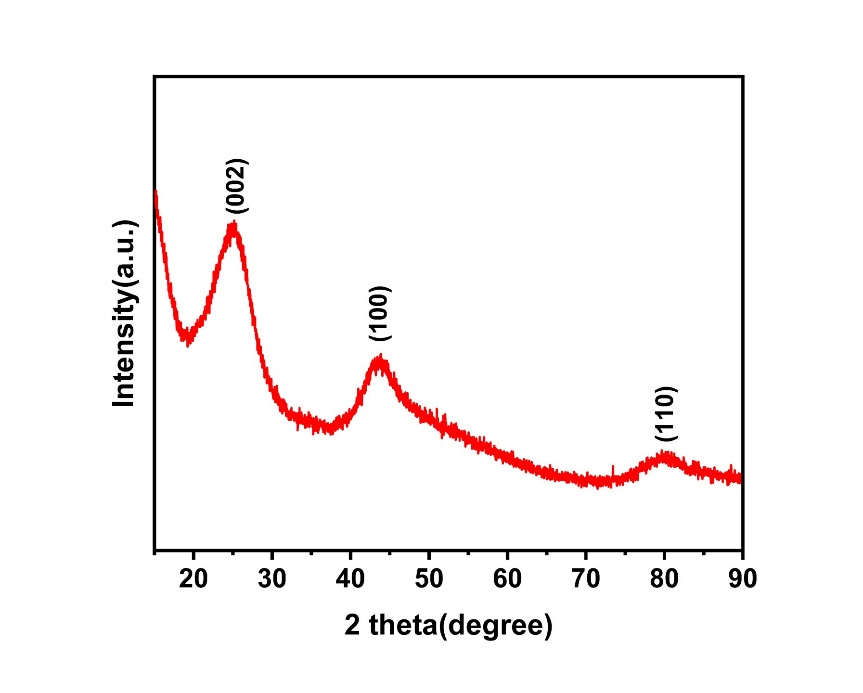


**Figure S7.** XRD patterns of the carbonized skeleton.

Figure S7 shows a typical X-ray diffraction (XRD) pattern of pyrolytic carbon containing three broad peaks located at (002), (100), and (110) diffraction, respectively. ^[1-3]^ The peaks indicate that the pyrolytic carbon contains turbulent graphene layers. These findings suggest that a crystal is composed of multiple stacked graphite layers.


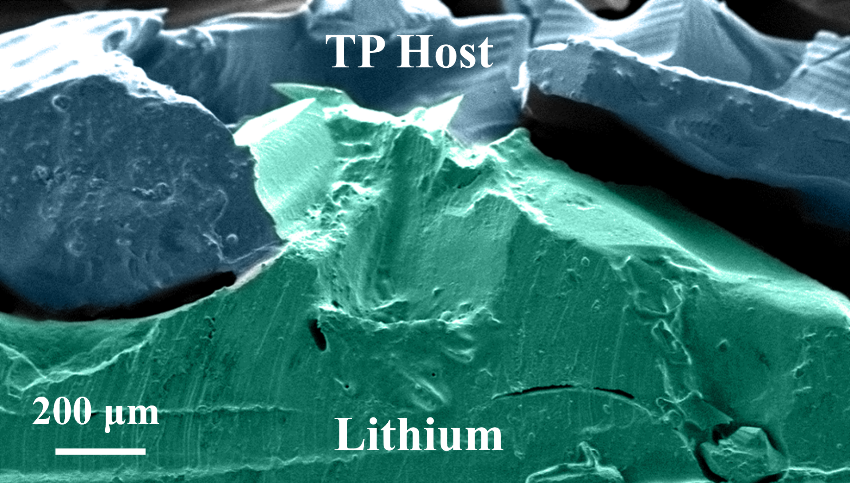


**Figure S8.** High-magnification cross-sectional SEM images of TP-Li after prolonged cycling at 20 mA cm⁻² and 20 mAh cm⁻².

The topology-optimized framework remains structurally intact after cycling, with no observable large-scale fracture or collapse of the reinforcing ribs. Lithium is observed to be confined within the internal architecture of the host, indicating effective structural accommodation of volume variation during repeated plating/stripping processes.

**Table S1**. Geometric Parameters of Topology and Mesh Structures in Finite Element Analysis.

| Entire structure geometric parameters | Values |
| --- | --- |
| Unit Length (mm) | 10 |
| Unit Width (mm) | 10 |
| Unit Height (mm) | 10 |
| Mesh Size (mm) | 0.01 |

**Table S2**. Printing parameters for the host skeleton.

| Printing parameters |  |
| --- | --- |
| Layer Height (mm) | 0.05 |
| Base Time (s) | 3 |
| Attach Time (s) | 10 |
| Gradual Time Layers | 4 |
| Light Intensity | 100% |

**Table S3.** Parameters in the mechano-electrochemical model of COMSOL.

| Parameters | Value |
| --- | --- |
| The initial concentration of electrolyte (mol m^-3^) | 1000 |
| Electrical conductivity of the electrode (S m^-1^) | 1 |
| Young's modulus of the electrode (GPa) | 2 |
| Poisson's ratio of the electrode | 0.34 |
| Electrical conductivity of electrolyte (S m^-1^) | 1.147 |
| Young's modulus of the 3D-printed Skeleton (GPa) | 2636 |
| Poisson's ratio of the 3D-printed Skeleton | 0.3 |

**References**

1. P. Trucano and R. Chen, "Structure of graphite by neutron diffraction," *Nature,* vol. 258, no. 5531, pp. 136-137, 1975/11/01 1975, https://doi: 10.1038/258136a0.

2. Z. Q. Li, C. J. Lu, Z. P. Xia, Y. Zhou, and Z. Luo, "X-ray diffraction patterns of graphite and turbostratic carbon," *Carbon,* vol. 45, no. 8, pp. 1686-1695, 2007/07/01/ 2007, https://doi.org/10.1016/j.carbon.2007.03.038.

3. A. Cuesta, P. Dhamelincourt, J. Laureyns, A. Martínez-Alonso, and J. M. D. Tascón, "Comparative performance of X-ray diffraction and Raman microprobe techniques for the study of carbon materials," *Journal of Materials Chemistry,* vol. 8, no. 12, pp. 2875-2879, 1998, https://doi: 10.1039/A805841E.
